# Supplementary material for: Transcriptomic profiling of unmethylated full mutation carriers implicates TET3 in FMR1 CGG repeat expansion methylation dynamics in fragile X syndrome
Source: J Neurodev Disord. 2025 Apr 26;17:22. doi: 10.1186/s11689-025-09609-5 (PMC12032669; doi:10.1186/s11689-025-09609-5)
Supplement: Supplementary file 1 — Supplementary Material 1 [file 11689_2025_9609_MOESM1_ESM.pdf]

CxxC domain

```
CLUSTAL O(1.2.4) multiple sequence alignment


Mouse_ENSMUST00000186548.7        MSQFQVPLAVQPDLSGLYDFPQGQVMVGGFQGPGLPMAGSETQLRGGGDGRKKRKRCGTC 60
Human_ENST00000409262.8           MSQFQVPLAVQPDLPGLYDFPQRQVMVGSFPGSGLSMAGSESQLRGGGDGRKKRKRCGTC 60
Rhesus_ENSMMUT00000028918.4       MSQFQVPLAVQPDLPGLYDFPQGQVMVGGFPGPELSMAGSESQLRGGGDGRKKRKRCGTC 60
Rhesus_ENSMMUT00000028916.3       MSQFQVPLAVQPDLPGLYDFPQGQVMVGGFPGPELSMAGSESQLRGGGDGRKKRKRCGTC 60
Rhesus_ENSMMUT00000075204.2       MSQFQVPLAVQPDLPGLYDFPQGQVMVGGFPGPELSMAGSESQLRGGGDGRKKRKRCGTC 60
                                  ************** ******* *****.* *  * *****:******************

Mouse_ENSMUST00000186548.7        DPCRRLENCGSCTSCTNRRTHQICKLRKCEVLKKKAGLLKEVEINAREGTGPWAQGATVK 120
Human_ENST00000409262.8           EPCRRLENCGACTSCTNRRTHQICKLRKCEVLKKKVGLLKEVEIKAGEGAGPWGQGAAVK 120
Rhesus_ENSMMUT00000028918.4       EPCRRLENCGACTSCTNRRTHQICKLRKCEVLKKKVGLLKEVEIKAGEGAGPWGQGAAVK 120
Rhesus_ENSMMUT00000028916.3       EPCRRLENCGACTSCTNRRTHQICKLRKCEVLKKKVGLLKEVEIKAGEGAGPWGQGAAVK 120
Rhesus_ENSMMUT00000075204.2       EPCRRLENCGACTSCTNRRTHQICKLRKCEVLKKKVGLLKE------------------- 101
                                  :*********:************************.*****

Mouse_ENSMUST00000186548.7        TGSELSPVDGPVPGQMDSGPVYHGDSRQLSTSGAPVNGAREPAGPGLLGAAGPWRVDQKP 180
Human_ENST00000409262.8           TGSELSPVDGPVPGQMDSGPVYHGDSRQLSASGVPVNGAREPAGPSLLGTGGPWRVDQKP 180
Rhesus_ENSMMUT00000028918.4       TGSELSPVDGPVPGQMDSGPVYHGDSRQLSASGVPVNGAREPAGPSLLGTGGPWRVDQKP 180
Rhesus_ENSMMUT00000028916.3       TGSELSPVDGPVPGQMDSGPVYHGDSRQLSASGVPVNGAREPAGPSLLGTGGPWRVDQKP 180
Rhesus_ENSMMUT00000075204.2       TGSELSPVDGPVPGQMDSGPVYHGDSRQLSASGVPVNGAREPAGPSLLGTGGPWRVDQKP 161
                                  ******************************:**.***********.***:.*********

Mouse_ENSMUST00000186548.7        DWEAASGPTHAARLEDAHDLVAFSAVAEAVSSYGALSTRLYETFNREMSREAGSNGRGPR 240
Human_ENST00000409262.8           DWEAAPGPAHTARLEDAHDLVAFSAVAEAVSSYGALSTRLYETFNREMSREAGNNSRGPR 240
Rhesus_ENSMMUT00000028918.4       DWEAAPGPAHTARLEDAHDLVAFSAVAEAVSSYGALSTRLYETFNREMSREAGNNSRGSR 240
Rhesus_ENSMMUT00000028916.3       DWEAAPGPAHTARLEDAHDLVAFSAVAEAVSSYGALSTRLYETFNREMSREAGNNSRGSR 240
Rhesus_ENSMMUT00000075204.2       DWEAAPGPAHTARLEDAHDLVAFSAVAEAVSSYGALSTRLYETFNREMSREAGNNSRGSR 221
                                  ***** **:*:******************************************.*.** *

Mouse_ENSMUST00000186548.7        --PESCSEGSEDLDTLQTALALARHGMKPPNCTCDGPECPDFLEWLEGKIKSMAMEGGQG 298
Human_ENST00000409262.8           PGPEGCSAGSEDLDTLQTALALARHGMKPPNCNCDGPECPDYLEWLEGKIKSVVMEGGEE 300
Rhesus_ENSMMUT00000028918.4       PGPEGCSAGSEDLDTLQTALALARH----------------------------------- 265
Rhesus_ENSMMUT00000028916.3       PGPEGCSAGSEDLDTLQTALALARHGMKPPNCNCDGPECPDYLEWLEGKIKSVVMEGGEE 300
Rhesus_ENSMMUT00000075204.2       PGPEGCSAGSEDLDTLQTALALARHGMKPPNCNCDGPECPDYLEWLEGKIKSVVMEGGEE 281
                                    **.** *****************

Mouse_ENSMUST00000186548.7        RPRLPGALPPSEAGLPAPSTRPPLLSSEVPQVPPLEGLPLSQSALSIAKEKNISLQTAIA 358
Human_ENST00000409262.8           RPRLPGPLPPGEAGLPAPSTRP-LLSSEVPQISPQEGLPLSQSALSIAKEKNISLQTAIA 359
Rhesus_ENSMMUT00000028918.4       ------------------------------------------------------------ 265
Rhesus_ENSMMUT00000028916.3       RPRLPGPLPPGEAGLPAPSTRP-LLSSEVPQISPQEGLPLSQSALSIAKEKNISLQTAIA 359
Rhesus_ENSMMUT00000075204.2       RPRLPGPLPPGEAGLPAPSTRP-LLSSEVPQISPQEGLPLSQSALSIAKEKNISLQTAIA 340


Mouse_ENSMUST00000186548.7        IEALTQLSSALPQPSHSTSQASCPLPEALSPSAPFRSPQSYLRAPSWPVVPPEEHPSFAP 418
Human_ENST00000409262.8           IEALTQLSSALPQPSHSTPQASCPLPEALSPPAPFRSPQSYLRAPSWPVVPPEEHSSFAP 419
Rhesus_ENSMMUT00000028918.4       ------------------------------------------------------------ 265
Rhesus_ENSMMUT00000028916.3       IEALTQLSSALPQPSHSTPQASCPLPEALSPPAPFRSPQSYLRAPSWPVVPPEEHSSFAP 419
Rhesus_ENSMMUT00000075204.2       IEALTQLSSALPQPSHSTPQASCPLPEALSPPAPFRSPQSYLRAPSWPVVPPEEHSSFAP 400


Mouse_ENSMUST00000186548.7        DSPAFPPATPRPEFSEAWGTDTPPATPRNSWPVPRPSPDPMAELEQLLGSASDYIQSVFK 478
Human_ENST00000409262.8           DSSAFPPATPRTEFPEAWGTDTPPATPRSSWPMPRPSPDPMAELEQLLGSASDYIQSVFK 479
Rhesus_ENSMMUT00000028918.4       -------ATPRTEFPEVWGTDTPPATPRSSWPMPRPSHDPMAELEQLLGSASDYIQSVFK 318
Rhesus_ENSMMUT00000028916.3       DSSAFPPATPRTEFPEVWGTDTPPATPRSSWPMPRPSHDPMAELEQLLGSASDYIQSVFK 479
Rhesus_ENSMMUT00000075204.2       DSSAFPPATPRTEFPEVWGTDTPPATPRSSWPMPRPSHDPMAELEQLLGSASDYIQSVFK 460
                                         **** ** *.***********.***:**** **********************

Mouse_ENSMUST00000186548.7        RPEALPTKPKVKVEAPSSSPAPVPSPISQREAPLLSSEPDTHQKAQTALQQHLHHKRNLF 538
Human_ENST00000409262.8           RPEALPTKPKVKVEAPSSSPAPAPSPVLQREAPTPSSEPDTHQKAQTALQQHLHHKRSLF 539
Rhesus_ENSMMUT00000028918.4       RPEALPTKPKVKVEAPSSSPALAPSPVLQREAPTPSSEPDTHQKAQTALQQHLHHKRSLF 378
Rhesus_ENSMMUT00000028916.3       RPEALPTKPKVKVEAPSSSPALAPSPVLQREAPTPSSEPDTHQKAQTALQQHLHHKRSLF 539
Rhesus_ENSMMUT00000075204.2       RPEALPTKPKVKVEAPSSSPALAPSPVLQREAPTPSSEPDTHQKAQTALQQHLHHKRSLF 520
                                  ********************* .***: *****  **********************.**

Mouse_ENSMUST00000186548.7        LEQAQDASFPTSTEPQAPGWWAPPGSPAPRPPDKPPKEKKKKPPTPAGGPVGAEKTTPGI 598
Human_ENST00000409262.8           LEQVHDTSFPAPSEPSAPGWWPPPSSPVPRLPDRPPKEKKKKLPTPAGGPVGTEKAAPGI 599
Rhesus_ENSMMUT00000028918.4       LEQAHDTSFPAPSEPSAPGWWPPPSSPAPRLPDRPPKEKKKKLPTPAGGPVGTEKAAPGI 438
Rhesus_ENSMMUT00000028916.3       LEQAHDTSFPAPSEPSAPGWWPPPSSPAPRLPDRPPKEKKKKLPTPAGGPVGTEKAAPGI 599
Rhesus_ENSMMUT00000075204.2       LEQAHDTSFPAPSEPSAPGWWPPPSSPAPRLPDRPPKEKKKKLPTPAGGPVGTEKAAPGI 580
                                  ***.:*:***: :**.***** **.**.** **:******** *********:**::***
```

```
Mouse_ENSMUST00000186548.7      KTSVRKPIQIKKSRSRDMQPLFLPVRQIVLEGLKPQASEGQAPLPAQLSVPPPASQGAAS    658
Human_ENST00000409262.8         KPSVRKPIQIKKSRPREAQPLFPPVRQIVLEGLRSPASQEVQAHP---PAPL-----PAS    651
Rhesus_ENSMMUT00000028918.4     KPSVRKPIQIKKSRPREAQPLFPPVRQIVLEGLRSPASQEVQAHP---PAPL-----PAS    490
Rhesus_ENSMMUT00000028916.3     KPSVRKPIQIKKSRPREAQPLFPPVRQIVLEGLRSPASQEVQAHP---PAPL-----PAS    651
Rhesus_ENSMMUT00000075204.2     KPSVRKPIQIKKSRPREAQPLFPPVRQIVLEGLRSPASQEVQAHP---PAPL-----PAS    632
                                * ************ *: **** **********:  **:     *    .*       **

Mouse_ENSMUST00000186548.7      QSCATPLTPEPSLALFAPSPSGDSLLPPTQEMRSPSPMVALQSGSTGGPLPPADDKLEEL    718
Human_ENST00000409262.8         QGSAVPLPPEPSLALFAPSPSRDSLLPPTQEMRSPSPMTALQPGST-GPLPPADDKLEEL    710
Rhesus_ENSMMUT00000028918.4     QGSAVPLPPEPSLALFAPSPSRDSLLPPTQEMRSPSPMTTLQPGST-GPLPPADDKLEEL    549
Rhesus_ENSMMUT00000028916.3     QGSAVPLPPEPSLALFAPSPSRDSLLPPTQEMRSPSPMTTLQPGST-GPLPPADDKLEEL    710
Rhesus_ENSMMUT00000075204.2     QGSAVPLPPEPSLALFAPSPSRDSLLPPTQEMRSPSPMTTLQPGST-GPLPPADDKLEEL    691
                                *..*.** ************* ****************.:** *** ************

Mouse_ENSMUST00000186548.7      IRQFEAEFGDSFGLPGPPSVPIQEPENQSTCLPAPESPFATRSPKKIKIESSGAVTVLST    778
Human_ENST00000409262.8         IRQFEAEFGDSFGLPGPPSVPIQDPENQQTCLPAPESPFATRSPKQIKIESSGAVTVLST    770
Rhesus_ENSMMUT00000028918.4     IRQFEAEFGDSFGLPGPPSVPIQDPENQQTCLPAPESPFATRSPKQIKIESSGAVTVLST    609
Rhesus_ENSMMUT00000028916.3     IRQFEAEFGDSFGLPGPPSVPIQDPENQQTCLPAPESPFATRSPKQIKIESSGAVTVLST    770
Rhesus_ENSMMUT00000075204.2     IRQFEAEFGDSFGLPGPPSVPIQDPENQQTCLPAPESPFATRSPKQIKIESSGAVTVLST    751
                                ***********************:****.****************:**************

Mouse_ENSMUST00000186548.7      TCFHSEEGGQEATPTKAENPLTPTLSGFLESPLKYLDTPTKSLLDTPAKKAQSEFPTCDC    838
Human_ENST00000409262.8         TCFHSEEGGQEATPTKAENPLTPTLSGFLESPLKYLDTPTKSLLDTPAKRAQAEFPTCDC    830
Rhesus_ENSMMUT00000028918.4     TCFHSEEGGQEATPTKAENPLTPTLSGFLESPLKYLDTPTKSLLDTPAKRAQAEFPTCDC    669
Rhesus_ENSMMUT00000028916.3     TCFHSEEGGQEATPTKAENPLTPTLSGFLESPLKYLDTPTKSLLDTPAKRAQAEFPTCDC    830
Rhesus_ENSMMUT00000075204.2     TCFHSEEGGQEATPTKAENPLTPTLSGFLESPLKYLDTPTKSLLDTPAKRAQAEFPTCDC    811
                                *************************************************:**:*******

Mouse_ENSMUST00000186548.7      VEQIVEKDEGPYYTHLGSGPTVASIRELMEDRYGEKGKAIRIEKVIYTGKEGKSSRGCPI    898
Human_ENST00000409262.8         VEQIVEKDEGPYYTHLGSGPTVASIRELMEERYGEKGKAIRIEKVIYTGKEGKSSRGCPI    890
Rhesus_ENSMMUT00000028918.4     VEQIVEKDEGPYYTHLGSGPTVASIRELMEERYGEKGKAIRIEKVIYTGKEGKSSRGCPI    729
Rhesus_ENSMMUT00000028916.3     VEQIVEKDEGPYYTHLGSGPTVASIRELMEERYGEKGKAIRIEKVIYTGKEGKSSRGCPI    890
Rhesus_ENSMMUT00000075204.2     VEQIVEKDEGPYYTHLGSGPTVASIRELMEERYGEKGKAIRIEKVIYTGKEGKSSRGCPI    871
                                ******************************:*****************************

Mouse_ENSMUST00000186548.7      AKWVIRRHTLEEKLLCLVRHRAGHHCQNAVIVILILAWEGIPRSLGDTLYQELTDTLRKY    958
Human_ENST00000409262.8         AKWVIRRHTLEEKLLCLVRHRAGHHCQNAVIVILILAWEGIPRSLGDTLYQELTDTLRKY    950
Rhesus_ENSMMUT00000028918.4     AKWVIRRHTLEEKLLCLVRHRAGHHCQNAVIVILILAWEGIPRSLGDTLYQELTDTLRKY    789
Rhesus_ENSMMUT00000028916.3     AKWVIRRHTLEEKLLCLVRHRAGHHCQNAVIVILILAWEGIPRSLGDTLYQELTDTLRKY    950
Rhesus_ENSMMUT00000075204.2     AKWVIRRHTLEEKLLCLVRHRAGHHCQNAVIVILILAWEGIPRSLGDTLYQELTDTLRKY    931
                                ************************************************************

Mouse_ENSMUST00000186548.7      GNPTSRRCGLNDDRTCACQGKDPNTCGASFSFGCSWSMYFNGCKYARSKTPRKFRLTGDN    1018
Human_ENST00000409262.8         GNPTSRRCGLNDDRTCACQGKDPNTCGASFSFGCSWSMYFNGCKYARSKTPRKFRLAGDN    1010
Rhesus_ENSMMUT00000028918.4     GNPTSRRCGLNDDRTCACQGKDPNTCGASFSFGCSWSMYFNGCKYARSKTPRKFRLAGDN    849
Rhesus_ENSMMUT00000028916.3     GNPTSRRCGLNDDRTCACQGKDPNTCGASFSFGCSWSMYFNGCKYARSKTPRKFRLAGDN    1010
Rhesus_ENSMMUT00000075204.2     GNPTSRRCGLNDDRTCACQGKDPNTCGASFSFGCSWSMYFNGCKYARSKTPRKFRLAGDN    991
                                ********************************************************:***

Mouse_ENSMUST00000186548.7      PKEEEVLRNSFQDLATEVAPLYKRLAPQAYQNQVTNEDVAIDCRLGLKEGRPFSGVTACM    1078
Human_ENST00000409262.8         PKEEEVLRKSFQDLATEVAPLYKRLAPQAYQNQVTNEEIAIDCRLGLKEGRPFAGVTACM    1070
Rhesus_ENSMMUT00000028918.4     PKEEEVLRKSFQDLATEVAPLYKRLAPQAYQNQVTNEEIAIDCRLGLKEGRPFAGVTACM    909
Rhesus_ENSMMUT00000028916.3     PKEEEVLRKSFQDLATEVAPLYKRLAPQAYQNQVTNEEIAIDCRLGLKEGRPFAGVTACM    1070
Rhesus_ENSMMUT00000075204.2     PKEEEVLRKSFQDLATEVAPLYKRLAPQAYQNQVTNEEIAIDCRLGLKEGRPFAGVTACM    1051
                                ********:****************************::**************:******

Mouse_ENSMUST00000186548.7      DFCAHAHKDQHNLYNGCTVVCTLTKEDNRCVGQIPEDEQLHVLPLYKMASTDEFGSEENQ    1138
Human_ENST00000409262.8         DFCAHAHKDQHNLYNGCTVVCTLTKEDNRCVGKIPEDEQLHVLPLYKMANTDEFGSEENQ    1130
Rhesus_ENSMMUT00000028918.4     DFCAHAHKDQHNLYNGCTVVCTLTKEDNRCVGKIPEDEQLHVLPLYKMASTDEFGSEENQ    969
Rhesus_ENSMMUT00000028916.3     DFCAHAHKDQHNLYNGCTVVCTLTKEDNRCVGKIPEDEQLHVLPLYKMASTDEFGSEENQ    1130
Rhesus_ENSMMUT00000075204.2     DFCAHAHKDQHNLYNGCTVVCTLTKEDNRCVGKIPEDEQLHVLPLYKMASTDEFGSEENQ    1111
                                ********************************:****************.**********

Mouse_ENSMUST00000186548.7      NAKVSSGAIQVLTAFPREVRRLPEPAKSCRQRLEARKAAAEKKKLQKEKLSTPEKIKQE    1198
Human_ENST00000409262.8         NAKVGSGAIQVLTAFPREVRRLPEPAKSCRQRLEARKAAAEKKKIQKEKLSTPEKIKQE    1190
Rhesus_ENSMMUT00000028918.4     NAKVGSGAIQVLTAFPREVRRLPEPAKSCRQRLEARKAAAEKKKIQKEKLSTPEKIKQE    1029
Rhesus_ENSMMUT00000028916.3     NAKVGSGAIQVLTAFPREVRRLPEPAKSCRQRLEARKAAAEKKKIQKEKLSTPEKIKQE    1190
Rhesus_ENSMMUT00000075204.2     NAKVGSGAIQVLTAFPREVRRLPEPAKSCRQRLEARKAAAEKKKIQKEKLSTPEKIKQE    1171
                                ****.***************************************:***************

Mouse_ENSMUST00000186548.7      ALELAGVTTDPGLSLKGGLSQQSLKPSLKVEPQNHFSSFKYSGNAVVESYSVLGSCRPSD    1258
Human_ENST00000409262.8         ALELAGITSDPGLSLKGGLSQQGLKPSLKVEPQNHFSSFKYSGNAVVESYSVLGNCRPSD    1250
Rhesus_ENSMMUT00000028918.4     ALELAGVTSDPGLSLKGGLSQQGLKPSLKVEPQNHFSSFKYSSNAVVESYSVLGNCRPSD    1089
Rhesus_ENSMMUT00000028916.3     ALELAGVTSDPGLSLKGGLSQQGLKPSLKVEPQNHFSSFKYSSNAVVESYSVLGNCRPSD    1250
Rhesus_ENSMMUT00000075204.2     ALELAGVTSDPGLSLKGGLSQQGLKPSLKVEPQNHFSSFKYSSNAVVESYSVLGNCRPSD    1231
                                ******:*:*************.*******************.***********.*****

Mouse_ENSMUST00000186548.7      PYSMSSVYSYHSRYAQPGLASVNGFHSKYTLPSFGYYGFPSSNPVFPSQFLGPSAWGHGG    1318
Human_ENST00000409262.8         PYSMNSVYSYHSYYAQPSLTSVNGFHSKYALPSFSYYGFPSSNPVFPSQFLGPGAWGHSG    1310
Rhesus_ENSMMUT00000028918.4     PYSMNSVYSYHSYYAQPSLTSVNGFHSKYALPSFGYYGFPSSNPVFPSQFLGPGAWGHSG    1149
Rhesus_ENSMMUT00000028916.3     PYSMNSVYSYHSYYAQPSLTSVNGFHSKYALPSFGYYGFPSSNPVFPSQFLGPGAWGHSG    1310
Rhesus_ENSMMUT00000075204.2     PYSMNSVYSYHSYYAQPSLTSVNGFHSKYALPSFGYYGFPSSNPVFPSQFLGPGAWGHSG    1291
                                ****.******* ****.*:*********:****.******************.****.*
```

CYS rich | Catalytic residue | Large low complexity insert

```
Mouse_ENSMUST00000186548.7      SGGSFEKKPDLHALHNSLNPAYGGAEFAELPGQAVATDNHHPIPHHQQPAYPGPKEYLLP      1378
Human_ENST00000409262.8         SSGSFEKKPDLHALHNSLSPAYGGAEFAELPSQAVPTDAHHPTPHHQQPAYPGPKEYLLP      1370
Rhesus_ENSMMUT00000028918.4     SSGSFEKKPDLHALHNSLSPAYGGAEFAELPSQAVPTDAHHPTPHHQQPAYPGPKEYLLP      1209
Rhesus_ENSMMUT00000028916.3     SSGSFEKKPDLHALHNSLSPAYGGAEFAELPSQAVPTDAHHPTPHHQQPAYPGPKEYLLP      1370
Rhesus_ENSMMUT00000075204.2     SSGSFEKKPDLHALHNSLSPAYGGAEFAELPSQAVPTDAHHPTPHHQQPAYPGPKEYLLP      1351
                                *.****************.************.*** ** *** *****************

Mouse_ENSMUST00000186548.7      KVPQLHPASRDPSPFAQSSSCYNRSIKQEPIDPLTQAESIPRDSAKMSRTPLPEASQNGG      1438
Human_ENST00000409262.8         KAPLLHSVSRDPSPFAQSSNCYNRSIKQEPVDPLTQAEPVPRDAGKMGKTPLSEVSQNGG      1430
Rhesus_ENSMMUT00000028918.4     KAPPIHSVSRDPSPFAQSSNCYNRSIKQEPVDPLTQAEPVPRDAGKMGKTPLPEVSQNGG      1269
Rhesus_ENSMMUT00000028916.3     KAPPIHSVSRDPSPFAQSSNCYNRSIKQEPVDPLTQAEPVPRDAGKMGKTPLPEVSQNGG      1430
Rhesus_ENSMMUT00000075204.2     KAPPIHSVSRDPSPFAQSSNCYNRSIKQEPVDPLTQAEPVPRDAGKMGKTPLPEVSQNGG      1411
                                *.* :* .***********.**********:******* :***:.**.:*** *.*****

Mouse_ENSMUST00000186548.7      PSHLWGQYSGGPSMSPKRTNSVGGNWGVFPPGESPTIVPDKLNSFGASCLTPSHFPESQW      1498
Human_ENST00000409262.8         PSHLWGQYSGGPSMSPKRTNGVGGSWGVFSSGESPAIVPDKLSSFGASCLAPSHFTDGQW      1490
Rhesus_ENSMMUT00000028918.4     PSHLWGQYSGGPSMSPKRTNSVGGSWGVFSSGESPALIPDKLSSFGASCLAPSHFTDGQW      1329
Rhesus_ENSMMUT00000028916.3     PSHLWGQYSGGPSMSPKRTNSVGGSWGVFSSGESPALIPDKLSSFGASCLAPSHFTDGQW      1490
Rhesus_ENSMMUT00000075204.2     PSHLWGQYSGGPSMSPKRTNSVGGSWGVFSSGESPALIPDKLSSFGASCLAPSHFTDGQW      1471
                                ********************.***.****  ****:::****.*******:**** :.**

Mouse_ENSMUST00000186548.7      GLFTGEGQQSAPHAGARLRGKPWSPCKFGNGTSALTGPSLTEKPWGMGTGDFNPALKGGP      1558
Human_ENST00000409262.8         GLFPGEGQQAASHSGGRLRGKPWSPCKFGNSTSALAGPSLTEKPWALGAGDFNSALKGSP      1550
Rhesus_ENSMMUT00000028918.4     GLFPGEGQQAASHSGGRLRGKPWSPCKFGNSTSTLTGPGLTEKPWGLGAGDFNSALKGGP      1389
Rhesus_ENSMMUT00000028916.3     GLFPGEGQQAASHSGGRLRGKPWSPCKFGNSTSTLTGPGLTEKPWGLGAGDFNSALKGGP      1550
Rhesus_ENSMMUT00000075204.2     GLFPGEGQQAASHSGGRLRGKPWSPCKFGNSTSTLTGPGLTEKPWGLGAGDFNSALKGGP      1531
                                *** *****:* *:*.**************.**:*:**.******.:*:**** ****.*

Mouse_ENSMUST00000186548.7      GFQDKLWNPVKVEEGRIPTPGANPLDKAWQAFGMPLSSNEKLFGALKSEEKLWDPFSLEE      1618
Human_ENST00000409262.8         GFQDKLWNPMKGEEGRIPAAGASQLDRAWQSFGLPLGSSEKLFGALKSEEKLWDPFSLEE      1610
Rhesus_ENSMMUT00000028918.4     GFQDKLWNPMKGEEGRIPAAGASQLDKAWQSFGLSLGSSEKLFGALKSEEKLWDPFSLEE      1449
Rhesus_ENSMMUT00000028916.3     GFQDKLWNPMKGEEGRIPAAGASQLDKAWQSFGLSLGSSEKLFGALKSEEKLWDPFSLEE      1610
Rhesus_ENSMMUT00000075204.2     GFQDKLWNPMKGEEGRIPAAGASQLDKAWQSFGLSLGSSEKLFGALKSEEKLWDPFSLEE      1591
                                *********:* ******: **. **:***:**: *.*.*********************

Mouse_ENSMUST00000186548.7      GTAEEPPSKGVVKEEKSGPTVEEDEEELWSDSEHNFLDENIGGVAVAPAHCSILIECARR      1678
Human_ENST00000409262.8         GPAEEPPSKGAVKEEKGGGGAEEEEEELWSDSEHNFLDENIGGVAVAPAHGSILIECARR      1670
Rhesus_ENSMMUT00000028918.4     GPAEEPPSKGAVKEEKGGGGAEEEEEELWSDSEHNFLDENIGGVAVAPAHGSILIECARR      1509
Rhesus_ENSMMUT00000028916.3     GPAEEPPSKGAVKEEKGGGGAEEEEEELWSDSEHNFLDENIGGVAVAPAHGSILIECARR      1670
Rhesus_ENSMMUT00000075204.2     GPAEEPPSKGAVKEEKGGGGAEEEEEELWSDSEHNFLDENIGGVAVAPAHGSILIECARR      1651
                                * ********.*****.*  .**:************************** *********

Mouse_ENSMUST00000186548.7      ELHATTPLKKPNRCHPTRISLVFYQHKNLNQPNHGLALWEAKMKQLAERARQRQEEAARL      1738
Human_ENST00000409262.8         ELHATTPLKKPNRCHPTRISLVFYQHKNLNQPNHGLALWEAKMKQLAERARARQEEAARL      1730
Rhesus_ENSMMUT00000028918.4     ELHATTPLKKPNRCHPTRISLVFYQHKNLNQPNHGLALWEAKMKQLAERARARQEEAARL      1569
Rhesus_ENSMMUT00000028916.3     ELHATTPLKKPNRCHPTRISLVFYQHKNLNQPNHGLALWEAKMKQLAERARARQEEAARL      1730
Rhesus_ENSMMUT00000075204.2     ELHATTPLKKPNRCHPTRISLVFYQHKNLNQPNHGLALWEAKMKQLAERARARQEEAARL      1711
                                *************************************************** ********

Mouse_ENSMUST00000186548.7      GLGQQEAKLYGKKRKWGGAMVAEPQHKEKKGAIPTRQALAMPTDSAVTVSSYAYTKVTGP      1798
Human_ENST00000409262.8         GLGQQEAKLYGKKRKWGGTVVAEPQQKEKKGVVPTRQALAVPTDSAVTVSSYAYTKVTGP      1790
Rhesus_ENSMMUT00000028918.4     GLGQQEAKLYGKKRKWGGAMVAEPQQKEKKGVVPTRQALAVPTDSAVTVSSYAYTKVTGP      1629
Rhesus_ENSMMUT00000028916.3     GLGQQEAKLYGKKRKWGGAMVAEPQQKEKKGVVPTRQALAVPTDSAVTVSSYAYTKVTGP      1790
Rhesus_ENSMMUT00000075204.2     GLGQQEAKLYGKKRKWGGAMVAEPQQKEKKGVVPTRQALAVPTDSAVTVSSYAYTKVTGP      1771
                                ******************::*****:*****.:*******:*******************

Mouse_ENSMUST00000186548.7      YSRWI*      1803
Human_ENST00000409262.8         YSRWI*      1795
Rhesus_ENSMMUT00000028918.4     YSRWI*      1634
Rhesus_ENSMMUT00000028916.3     YSRWI*      1795
Rhesus_ENSMMUT00000075204.2     YSRWI*      1776
                                ******
```

CYS rich region

Catalytic residue

Large low complexity insert
