# Supplementary material for: Transcriptomic profiling of unmethylated full mutation carriers implicates TET3 in FMR1 CGG repeat expansion methylation dynamics in fragile X syndrome
Source: J Neurodev Disord. 2025 Apr 26;17:22. doi: 10.1186/s11689-025-09609-5 (PMC12032669; doi:10.1186/s11689-025-09609-5)
Supplement: Supplementary file 3 — Supplementary Material 3 [file 11689_2025_9609_MOESM3_ESM.docx]

**Supplemental data**


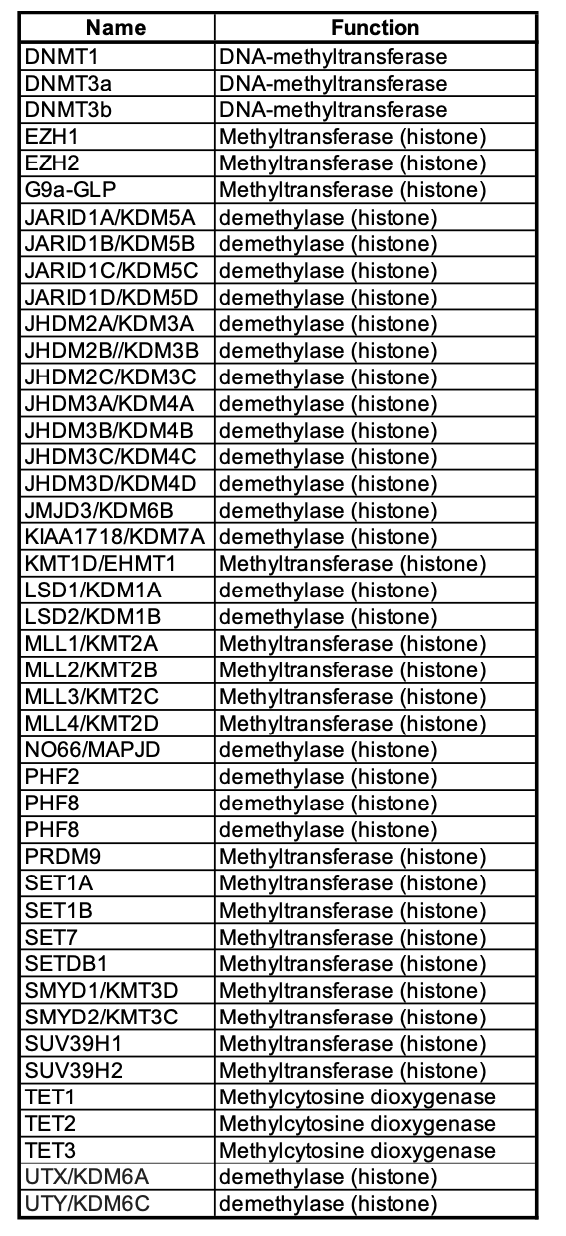


***Supplemental table 1***

*List of 44 candidate genes with roles in epigenetic modification of DNA*


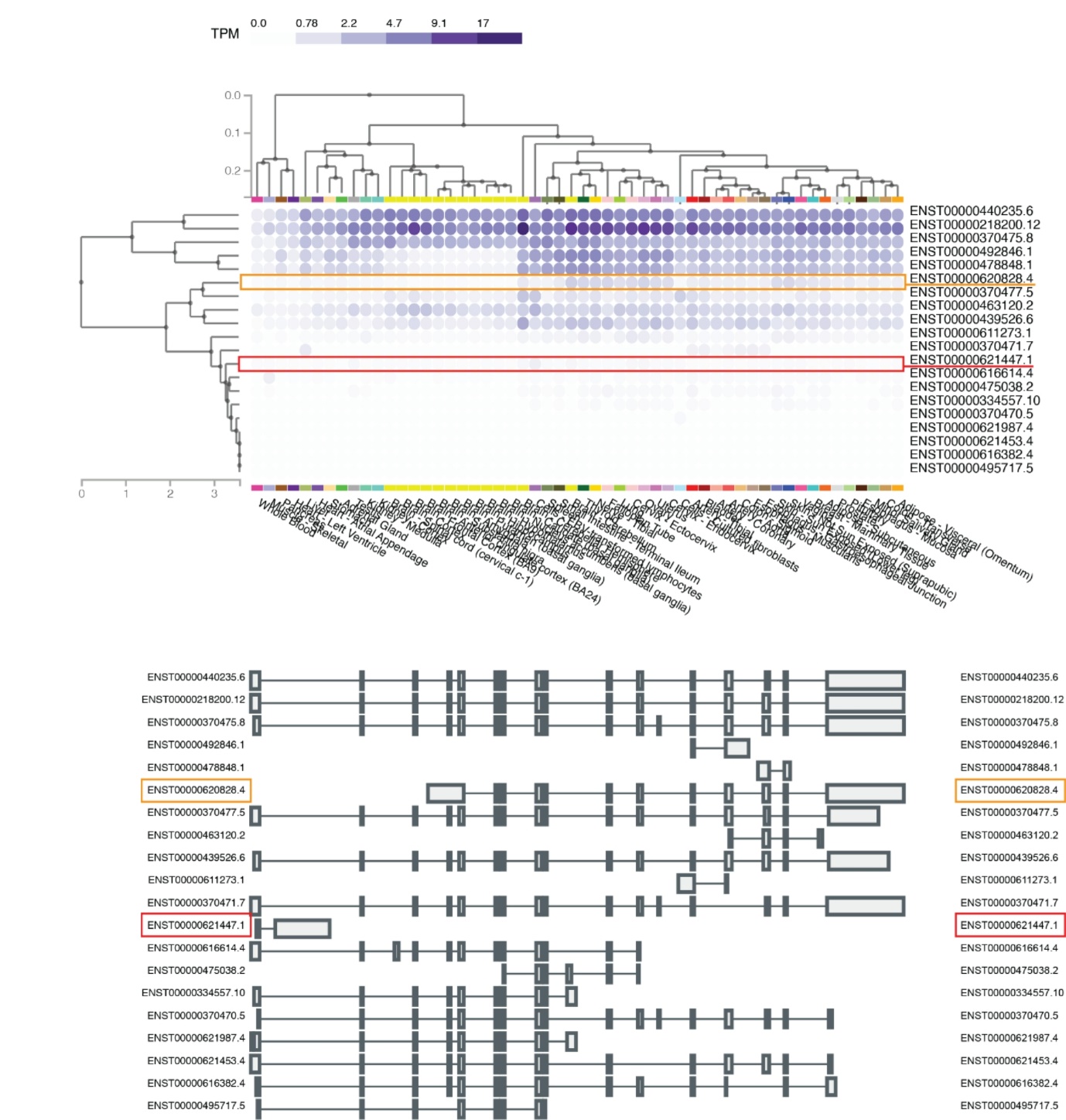


***Supplemental Fig. 1***

*FMR1 transcript information from gTEX*


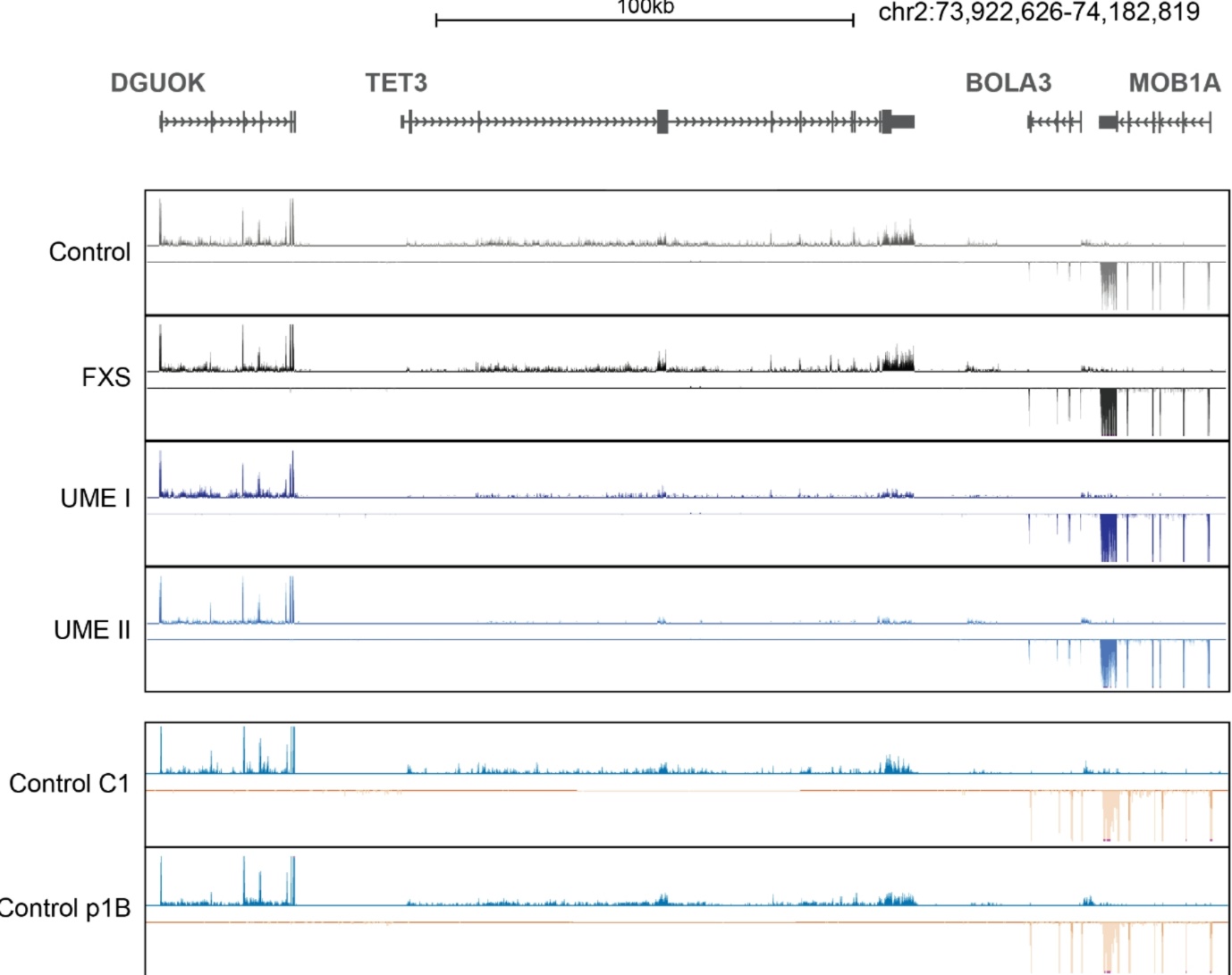


***Supplemental Fig. 2***

*Coverage plots of RNA-seq data at the TET3 gene from fibroblasts obtained from a control individual, an FXS patient and two FMR1 UFM carriers. Coverage tracks are scaled based on the total number of reads successfully mapped to the genome. Coverage tracks were generated from track collections and show merged tracks from 3 replicates. Control C1 and p1B showing RNA-seq coverage from unrelated fibroblast cell lines generated by Fernandes et al (jn prep) scaled to neighbouring genes.*
